# Educational Interventions for Promoting Food Literacy and Patient Engagement in Preventing Complications of Type 2 Diabetes: A Systematic Review

**DOI:** 10.3390/jpm11080795

**Published:** 2021-08-14

**Authors:** M. Savarese, M. Sapienza, G. M. Acquati, M. C. Nurchis, M. T. Riccardi, V. Mastrilli, R. D’Elia, E. A. Graps, G. Graffigna, G. Damiani

**Affiliations:** 1 EngageMinds HUB–Consumer Food & Health Engagement Research Center, Università Cattolica del Sacro Cuore, Largo A.Gemelli 1, 20123 Milano, Italy; mariarosaria.savarese@unicatt.it (M.S.); Guendalina.graffigna@unicatt.it (G.G.); 2 Faculty of Agriculture Food and Environmental Sciences, Università Cattolica del Sacro Cuore, 20123 Milano, Italy; 3 Department of Health Sciences and Public Health, Section of Hygiene, Università Cattolica del Sacro Cuore, 00168 Rome, Italy; martina.sapienza93@gmail.com (M.S.); gianfranco.damiani@unicatt.it (G.D.); 4 Faculty of Psychology, Università Cattolica del Sacro Cuore, 20123 Milano, Italy; gretamaria.acquati01@icatt.it; 5 Department of Woman and Child Health and Public Health, Fondazione Policlinico Universitario A.Gemelli IRCCS, Largo A.Gemelli 8, 00168 Rome, Italy; nurchismario@gmail.com; 6 Ministero Della Salute, Direzione Generale della Prevenzione, Ufficio 8-Promozione Salute e Prevenzione Controllo Malattie Cronico-Degenerative, 20123 Milano, Italy; v.mastrilli@sanita.it (V.M.); r.delia@sanita.it (R.D.); 7 A.Re.S.S. Puglia-Agenzia Regionale Strategica per la Salute ed il Sociale Area Valutazione e Ricerca, 20123 Milano, Italy; e.graps@aress.regione.puglia.it; 8 Department of Psychology, Università Cattolica del Sacro Cuore, Largo A.Gemelli 1, 20123 Milano, Italy

**Keywords:** patient engagement, food literacy, diabetes intervention, chronic disease

## Abstract

The present review aims to map the current literature on educational interventions to promote food literacy in type 2 diabetes, with a particular focus on the concept of patient engagement. The systematic review was implemented on five databases with no restrictions on the publication year. The studies selected for the review were focused on patients with type 2 diabetes, ranging from 2003 to 2021 and published in 13 countries (44% USA). Thirty-three articles were analyzed. Twenty-seven articles targeted singular patients; fifteen articles conceptualized patient engagement as self-management. In seven articles, the provider is a multidisciplinary team. Twenty articles did not report a theoretical framework in the intervention development, and eleven did not use an intervention material. Twenty-six articles did not use a technology proxy. Outcome categories were narratively mapped into four areas: clinical, psychological, behavioral, and literacy. To date, most of the interventions are heterogeneous in the adopted methodology, measures, and outcomes considered. More attention should be given to the psychosocial characterization of patient engagement as well as the technological support. High-quality, randomized controlled trials and longitudinal studies are lacking and need to be conducted to verify the efficacy of these insights.

## 1. Introduction

Diabetes is a major public health concern that is approaching epidemic proportions globally [1]. About 422 million people worldwide have diabetes, and 1.6 million deaths are directly attributed to diabetes each year. The most common is the type 2 diabetes. In the past three decades, the prevalence of type 2 diabetes has risen dramatically in countries of all income levels [2].

There is substantial evidence that leading a healthy lifestyle, including following a healthy diet, achieving modest weight loss, and performing regular physical activity, can maintain healthy blood glucose levels and reduce the risk of complications of type 2 diabetes [3]. Indeed, the American Diabetes Association (ADA) published guidelines highlighting that self-management and education are crucial aspects of diabetes care allowing the optimization of metabolic control, the improvement of overall quality of life, and the prevention of acute and chronic complications [4]. Given its nature, primary care can be a valuable setting for preventing diabetes and its complications in at-risk populations because it is a patient’s primary point of contact with the health care system. Patients can be offered support by primary care health professionals (e.g., general practitioners, practice nurses) for prevention, such as screening and lifestyle advice, as well as monitoring health outcomes [5]. For these reasons, scholars have been studying how to educate and engage patients in effective behavioral change towards better health outcomes [6,7,8]. The concept of food literacy is recognized in the literature as a fundamental ingredient for the management of chronic diseases, such as type 2 diabetes [9]. This concept is defined in the literature as the ability to develop knowledge and skills in food management, and it is a multi-componential concept that includes several aspects [10]. In a recent review of the literature [11], authors systematized the various definitions of food literacy, identifying these constitutional components: food skills, food nutritional knowledge, self-efficacy and attitudes towards food, food and dietary behaviors, ecological factors (socio-cultural, influences, and eating practices). This multi-component nature was also highlighted in the review from 2017 by Truman and colleagues [12]. However, both scholars and institutions suggested that knowledge alone is not sufficient to sustain a behavioral change in disease management, but it is necessary to gain a broader perspective that considers patients’ psychosocial aspects and how they contribute to their engagement in the care [13,14,15,16]. Recently, the World Health Organization confirmed the support of a change in this direction with the Shanghai 2016 declaration [17] that promotes both health literacy and empowerment for individuals to enable their participation in managing their health. Over the past 50 years, an extensive body of literature has emerged describing several concepts of the relationship between patients and healthcare systems. In this perspective, the patients are considered as full members of the healthcare team [18] not only with their disease but also with their psychological uniqueness, values, and experience [12,19,20] as the human component of the care. For the patients, to assume an active role in disease management, it means to shift from being a passive user of the healthcare services to being an active partner, emotionally resilient, and behaviorally able to adjust medical advices to their own disease status [16,21,22]. In fact, people with high levels of engagement have been identified as more effective in enhancing behavioral change and in adhering to medical prescriptions [23,24] and in diabetes management [25] and in having an overall better quality of care.

To sum up, in the past decades, the shift towards a more multifaceted approach to patients with diabetes is challenging the public health sector to lever on the patients themselves as the key actors for implementing effective educational interventions. In this scenario, concepts related to patient engagement have been recognized as an essential topic to sustain type 2 diabetes disease-management and prevention behaviors. However, the relative newness of this concept and the fragmentation of articles applying it to food literacy educational interventions in the scientific debate urges for a systematization aimed at providing innovative insights. 

In line with these premises, the aim of this systematic review is to map educational intervention for patients with type 2 diabetes in order to promote food literacy, with a particular focus on patient engagement, and to discuss the results about disease complications’ prevention. 

## 2. Materials and Methods

The systematic review protocol was registered in 2020 in the international prospective register of systematic reviews. The registration number is CRD42020167938.

### 2.1. Literature Search Strategy

A Boolean search string was created using the elements of the PICO model (P, population/patient; I, intervention/indicator; C, comparator/control; and O, outcome) to search for relevant articles in the MEDLINE, Cochrane Library, Web of Science, EMBASE, and Scopus databases [26]. References of individual studies were also back-checked. Articles were retrieved from the inception of each database until April 2021. 

The following search terms were used:

1. Terms related to population: “diabetes mellitus type 2”, “diabetes mellitus II”, “type 2 diabetes”, “type 2 diabetic patients”;
2. Terms linked to intervention: “educational interventions”, “literacy program, “food education”, “food literacy”, “nutrition education”, “health literacy”; and
3. Terms related to measured outcomes: “assessment”, “output”, “measurement”, “evaluation”.

This systematic review was conducted according to PRISMA (Preferred Reporting Items for Systematic Reviews and Meta-Analyses) guidelines for the reporting of systematic reviews [27].

### 2.2. Inclusion Criteria

The inclusion of relevant studies was based on the following criteria: (1) researches explicitly involving adult patients with type 2 diabetes as a unique or secondary condition as the main target of the intervention; (2) studies describing educational interventions (intended as standardized protocols, programs, courses implemented face-to-face or online, with or without the direct presence of a healthcare professional, and/or delivered with the support of dedicated educational materials); (3) studies analyzing educational interventions explicitly addressing food literacy, nutritional literacy, and/or health literacy; (4) studies reporting a measured outcome (measured with direct or self-reported tools) describing a psychological and/or motivational improvement of the patients (potentially labeled as patient empowerment, self-management, patient involvement, adherence, and compliance; patients activation; quality of life) [28]; and (5) studies involving individual, dyadic, and/or group interventions either self-administrated or administered by a healthcare professional. We focused on primary studies reporting efficacy results. 

The inclusion was restricted by age of participants (i.e., >18 year old), English language only, and availability of full texts published in peer-reviewed journals. 

After removing duplicate results, four researchers (S.M., A.G.M., N.M.C., S.M.) independently screened the title and abstract to outline the most appropriate articles. Then, the four researchers performed a full-text screening of each article to determine eligibility. First, the four researchers screened a pull of 20 articles together, with the aim to fine-tune the screening process and solve eventual misalignments. Secondly, the four researchers independently read the abstracts and proceeded with the selection of the pertinent ones. During the screening process, the researchers solved any ambiguous situation or bias by discussing together the inclusion or exclusion of the article based on the eligibility criteria identified and their expertise on the topic.

**Figure 1:**
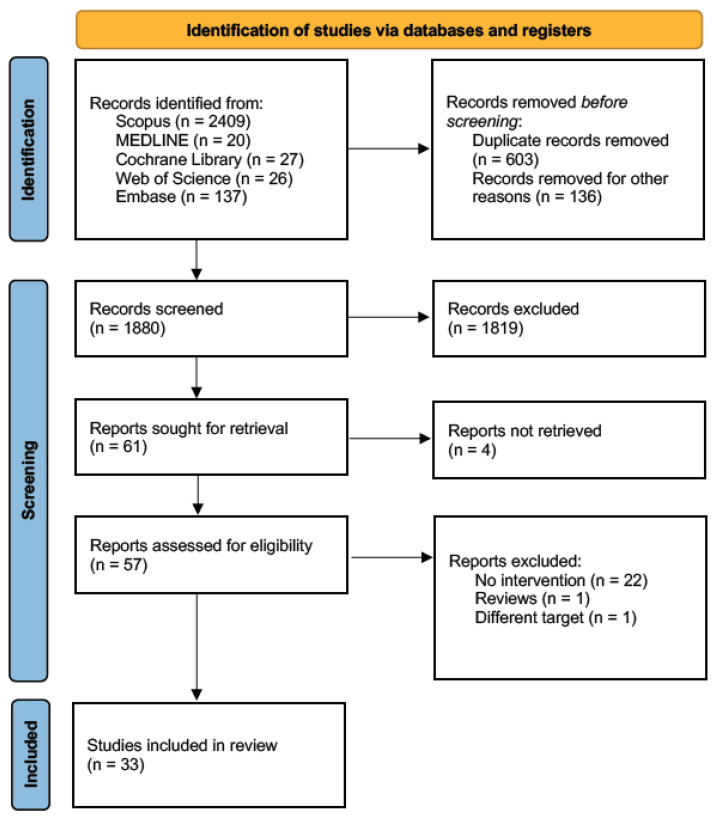
PRISMA flow diagram of different screening rounds.

The following PRISMA flow diagram reports the systematic review’s search and selection process of studies for inclusion (Figure 1).

### 2.3. Data Extraction and Quality Assessment

Data extraction was independently completed by four researchers (MS, GA, MCN, MS), adopting a standard data-entry electronic form. Data on study characteristics (i.e., author name, country or region of study, year of publication, study design), participants-related aspects (i.e., sample size, sex, age), intervention-related aspects (i.e., target and provider of intervention, duration of intervention, type and description of intervention, technology proxies, theoretical frameworks, conceptualization of patient engagement provided), and outcome-related aspects (i.e., outcome category and timing, outcome measurement tools) were extracted from each included study.

Study quality was measured using the Quality Criteria Checklist for primary research provided by the American Diabetes Association, assigning each study a rating of negative, neutral, or positive [29]. This checklist includes ten validity questions based on the Agency for Healthcare Research and Quality (AHRQ) domains for research studies. Sub-questions are listed under each validity question that identifies important aspects of sound study design and execution relevant to each domain. Some sub-questions also identify how the domain applies in specific research designs. Additionally, as established by the checklist guidelines, to address the heterogeneity of study designs, the several sub-questions were adapted to the specific study design of each eligible article. Moreover, to obtain a summary score for each validity question, a threshold was identified. In particular, if the answers to the sub-questions of each domain were “yes” in more than 50% of them, then the summary score was “yes”; if the answers to the sub-questions of each domain were “no” or “unclear” in 50% or more of them, then the summary score was “no”. Moreover, on the basis of the American Diabetes Association guidelines, the score was rated taking into account that, among the signaling questions, the numbers 2, 3, 6, and 7 (i.e., Was the selection of study subjects/patients free from bias?; Were study groups comparable?; Were intervention/therapeutic regimens/exposure factor or procedure and any comparison(s) described in detail? Were intervening factors described?; Were outcomes clearly defined and the measurements valid and reliable?) were deemed to be of higher importance with respect to the others.

### 2.4. Data Synthesis and Analysis 

The main features of the articles were extracted and described. Patient engagement was described in terms of the different conceptualizations and relative theoretical framework used. The level of food literacy and patient engagement were also analyzed since the expected significant variability in relation to different educational interventions. Results were analyzed and summarized narratively considering the methodological quality and scope of each study.

## 3. Results

### 3.1. Overview of the Studies 

After duplicates removal, a total of 1880 articles were retrieved from five databases; 1819 were excluded through title and abstract screening because they were not pertinent with the aims of the study, reported a different disease (i.e., type 1 diabetes, cardiovascular disease), or did not consider food literacy or patient-engagement outcomes. Twenty-eight articles were excluded after full-text analysis because they did not meet eligibility criteria (see Section 2.2). The articles included for the analyses ranged from 2003 to 2019 and were conducted in 13 different countries. The majority of the studies were conducted in the USA (n. 15) [30,31,32,33,34,35,36,37,38,39,40,41,42,43,44]; three in Iran [45,46,47] and Korea [8,48,49]; two in Canada [50,51] and the UK [52,53]; one in China [7], Belgium [6], Bulgaria [54], Hong Kong [55], Japan [56], Malaysia [57], Mexico [58], and Taiwan [59]. Considering articles’ design the majority (n. 21) was a Randomized Control Trial [7,8,30,32,33,34,35,36,37,38,39,41,45,47,48,49,51,52,53,57]; seven were a pre-post study [6,31,50,51,54,56,58]; three had a quasi-experimental design [43,55,59]; and one had a quasi-experimental case control [46]. The number of participants ranged from 17 to 1039 and had an average age between 43 and 74.5 (intervention sample). Table 1 reports an overview of all the included studies, describing year and country of the study; study design; outcome category; exposure timing; sample size (female; intervention and control); age (intervention and control) synthetic results; and long-term maintenance. 

Among the articles, narratively, different types of outcomes were grouped into broader categories: clinical outcomes (i.e., glycemic control, BMI, cholesterol, body pressure), behavioral outcomes (i.e., diet management, disease self-management, medications adherence, healthcare services utilization, physical activity), psychological (i.e., depression, quality of life, mental health in general, illness perception, patient satisfaction, patient activation, patient empowerment, self-efficacy, fatigue), and literacy (label-reading capabilities, knowledge).

Fourteen articles considered all these different types of outcomes together [6,30,31,32,34,38,40,44,49,50,52,54,57,59]; one article considered behavioral, psychological, and literacy outcomes [43]; one article considered clinical, behavioral, and psychological outcomes [49]; one article considered clinical, psychological, and literacy outcomes [41]; one article considered psychological and literacy outcomes [45]; one article considered behavioral and literacy outcomes [51]; six articles considered only clinical outcomes [33,35,39,48,56,58]; four articles considered only literacy [36,37,42,53]; and four articles considered only psychological outcomes [7,46,47,55].

Moreover, other studies described by this review considered aim of the intervention; intervention target; intervention provider; theory explicated; technology proxy involved; intervention materials; and outcome measure.

### 3.2. Quality Assessment 

Table 2 provides an overall risk score for the included studies. The majority of the studies (n = 25) were identified as neutral in rating quality. 

Twenty-one studies were rated negatively in the intervention/exposure validity question (i.e., Were intervention/therapeutic regimens/exposure factor or procedure and any comparison(s) described in detail? Were intervening factors described?). The vast majority of articles (n = 27) did not use blinding to prevent introduction of bias (i.e., Was blinding used to prevent introduction of bias?), while 27 studies did not describe methods of handling withdrawals (i.e., Was the method of handling withdrawals described?).

All the included articles conducted the most proper statistical analyses, while the majority (n = 28) of studies supported their conclusions taking into consideration biases and limitations. 

Signalling questions:

1. Was the research question clearly stated?
2. Was the selection of study subjects/patients free from bias?
3. Were study groups comparable?
4. Was method of handling withdrawals described?
5. Was blinding used to prevent introduction of bias?
6. Were intervention/therapeutic regimens/exposure factor or procedure and any comparison(s) described in detail? Were intervening factors described?
7. Were outcomes clearly defined and the measurements valid and reliable?
8. Was the statistical analysis appropriate for the study design and type of outcome indicators?
9. Are conclusions supported by results with biases and limitations taken into consideration?
10. Is bias due to study’s funding or sponsorship unlikely?

### 3.3. Outcome Categories

#### 3.3.1. Patient Engagement Components 

We classified the articles on the basis of the way patient engagement (as intended in this review) was conceptualized in the studies. In greater detail, fifteen articles conceptualized it as self-management alone [32,33,36,42,52,53] or together with other variables, such as quality of life, patient participation, or self-efficacy [6,8,35,38,40,44,47,48,51]. Seven articles described some kind of participation of the patients or the families into the definition or the adjustment of the intervention [30,31,34,37,41,46,54] in order to consider their opinion and to better target the intervention itself. Three articles included an evaluation of patient adherence to the treatment or the prescriptions [45,50,56] together with broader quality-of-life or empowerment measures. Three included the concept of patient activation [43,49,50]. Three included quality-of-life measures [55,58,59]. One used patient engagement [39] and one patient empowerment [7]. 

#### 3.3.2. Intervention Target 

The majority of the interventions (n = 27) described targeted individual patients [7,8,30,31,32,33,34,35,36,37,39,40,42,43,44,45,46,47,48,50,52,53,54,55,56,57]; four targeted patient groups [38,41,51,59]; and two both individuals and groups [6,58]. 

#### 3.3.3. Intervention Provider

Eight articles described the intervention provided by a multidisciplinary team [31,34,44,48,51,55,57,58] among the others, composed by endocrinologists, general practitioners, ophthalmologist, podiatrist nutritionists, nurses, educators, physical therapist or rehabilitation specialist, dietitian, psychologist, dermatologist, and dentist. Five articles were by a nurse, of which two were practicing alone [33,37], one under the supervision of a specialist [39], and two by a nurse specialized in diabetes education [36,49]. In five articles, the researchers provided the intervention itself [7,43,46,47,59]. Four articles were by lay workers [38,41,52,53]. Three articles were by educators [6,40], in one case alternatively to a health professional [6]. In three articles, the intervention was delivered by a pharmacist [35,54,56], and in the other three, the provider was not specified [8,32,45]. Finally, one article described the intervention as provided by a doctor [30] and one article by a coach [50]. 

#### 3.3.4. Theoretical Framework 

**Figure 2:**
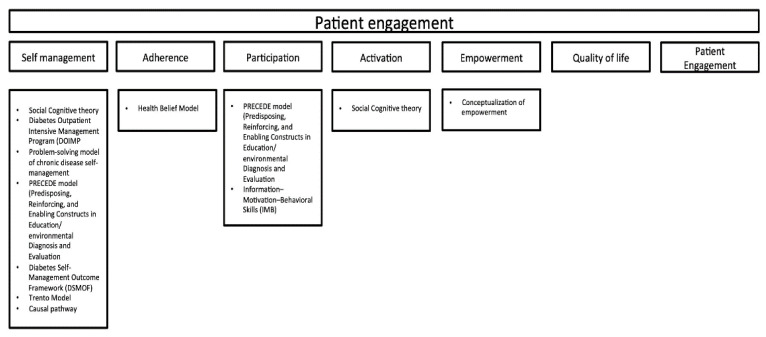
Graphical representation of the theoretical framework used for the different conceptualization of patient engagement.

Seventeen articles did not report a theoretical framework as the base for intervention development [31,33,35,37,39,42,43,47,50,52,53,54,55,56,57,58,59]. The other articles explained the theoretical framework or theory behind intervention development (n = 15). In particular, two articles reported the Social Cognitive theory inspired by Bandura [40,49,60]; one cited the Health-Belief Model [45,61]; and one the Trento Model by Trento and colleagues (2005) [51,62]; one article explicated theory related to self-efficacy in association with the Social Support Theory by Vaux (1998) [30,63] and two related to the concept of empowerment [41,59]; and one referred to the problem-solving model of chronic disease self-management by D’Zurilla and Nezu (1990) [32,64]. Finally, six articles framed the intervention in a more complex framework for behavioral change. Two of them referred to the PRECEDE model (Predisposing, Reinforcing, and Enabling Constructs in Education/Environmental Diagnosis and Evaluation [38,46] inspired by Lusk et al. [65]; one to the Information–Motivation–Behavioral Skills (IMB) [34,66]; one to the diabetes outpatient intensive management program (DOIMP) [48]; one to the causal pathway proposed by Fransen and colleagues (2012) [67]; and one to the Diabetes Self-Management Outcome Framework (DSMOF) [6]; and one article included a toolkit based on two previous validated models: the Diabetes Literacy and Numeracy Educational Toolkit and The American College of Physicians Foundation Living With Diabetes Guide [44]. We also crossed the theoretical framework with the conceptualization of patient engagement proposed by the different authors (Figure 2). 

#### 3.3.5. Intervention Materials 

Nine articles did not report or did not use any kind of intervention material [6,7,39,48,51,52,55,57,58].

Eight articles supported the intervention with a guide [33,43,44,45,46,49,50,53] in the form of a brochure, pamphlet, booklet, leaflet, among which one used together with films [46]. Four articles used visual materials, such as flipcharts alone [34] or in support of models and handouts [41], graphics and audio recordings [37] or conversation cards [59]. Three articles used a video [35,36,47], of which one used together with films, posters, and images [47]. Moreover, three articles used a questionnaire or checklist [40,54,56]: online [40] or in paper form [40,54,56]. Three articles used workbooks [8,32,42], one with the secondary materials (including a blood glucose meter, which measured and automatically transmitted results to a website; a rice bowl) [8]. Finally, one article used website information [30]; one article used multimedia materials [38]; and one article used only conversation maps [31]. 

#### 3.3.6. Technology Proxy

The majority of the articles (n = 24) did not use a technology proxy in the intervention [7,8,31,32,34,35,37,38,41,42,43,44,45,47,48,50,51,52,53,54,56,57,59]; the other three used the desktop computer or laptop as a tool to facilitate patients’ data transmission from the patient to the hospital [30,33,39]; three articles generically referred to the use of the Internet [6,30,47]; and one used social media [49], while two adopted emails and the hospital webpage as an informative tool [39,58]. 

#### 3.3.7. Outcome Measure

The outcome measures were classified based on the outcome category. Clinical measurements often occurred with standard techniques, so the measure tool was unspecified in most cases. A summary of the outcome categories and related measure tools are reported in Table 3. 

## 4. Discussion

The present systematic review mapped the educational interventions for type 2 diabetes patients aimed to promote food literacy, with a specific focus on patient engagement conceptualizations. All the interventions described in the included articles have highlighted how taking actions aimed at improving food literacy is a key element in achieving diabetes management [9].

Since patient education in type 2 diabetes is becoming more multifaceted and trying to integrate psychosocial aspects and literacy, scholars have published an increasing number of articles to investigate the effects of these variables on patients’ outcomes. This systematic review offers an integrated view on the phenomenon that categorizes the main features of the interventions and assesses the quality of the studies published to date. In greater detail, the articles included in this review ranged from 2003 to date, suggesting that scholars started to consider both aspects of food literacy and patient engagement only in the last two decades. The care of chronic diseases requires a deep reconfiguration of the patients’ life and the adaptation to a new lifestyle, which also encompasses disease management. For this reason, a more integrated approach to the education of these patients could have positive effects on both clinical [40,48,50] and psychosocial outcomes [46,55]. This appears to be in line with the conceptualization of patient-centered care proposed at the beginning of the new millennium [68]. This is also particularly relevant in the field of chronic diseases [69], such as diabetes. Overall, as highlighted in the latest literature [70], signals suggesting the increasing willingness of scholars to broaden the idea of diabetes education by approaching it from a multifaceted perspective were found. In our review, most of the articles conceptualized patient engagement in terms of self-management. Fewer studies included the idea of patients’ active participation in the development or fine-tuning of the intervention or to involve them in the decision making along the care journey. Even if these results could be interpreted as a first step towards the inclusion of patients as an active part of the care team, this idea is still conceptualized and limited to care management [36,47]. In line with this consideration, the theoretical frameworks mapped here also belong mainly to the self-management area. Patients’ ability to manage their care with awareness and specific skills is surely recognized as one of the primary goals of the care process [47]. However, recently, scholars called for a more integrated approach to patients in which they should be considered as a member of the team itself, with their behavioral and psychological resources [69]. The same emerged for the concept of food literacy, which was measured in the articles analyzed here as following more an operational definition rather than a multifactorial and social one. This appears to be in contrast with the recent literature that claims the need to overcome a vision of food literacy only aimed at filling patients’ knowledge gaps with information [71]. It now appears urgent to frame food literacy in a more subject-centered approach to literacy. 

Our systematic review also highlights a relevant involvement of the multidisciplinary team in the education interventions [38,51,55,58]. In line with the premises of this review, this result suggests that in the last years, the education of patients with type 2 diabetes involves different specialists able to work together to guarantee positive outcomes, as described by different authors from our work [51,58]. These results appear encouraging if framed in the recent literature that highlights how the support of different health professionals could be beneficial for the patients [72] and for the care team [73,74,75]. This is in accordance with the quadruple aim, which fosters both the enhancement of patients’ experience and the care-team wellbeing [76].

Our review further mapped that most of the studies adopted tools that were developed for the specific investigation being reported and did not use a validated theoretical framework [33,47,77]. The lack of theory-driven intervention could be discussed considering the difficulty to adapt specific educational objectives, which depends largely on the patients’ characteristics, such as literacy level, as discovered by Kim and colleagues (2019) [49], who found effective results in patients with lower initial literacy. However, the risk of not using a theory-driven intervention is that the results may remain fragmented without the possibility to guide other future research. 

Our systematic review also mapped the use of a technology proxy, which is nowadays recognized as an efficient support in boosting patients’ education, as already established by a previous research underlying that technological interventions could benefit people living with diabetes [78]. Only a few articles included a web tool (e.g., social media, web sites, apps) in their educational intervention [30,33,39]. However, it can be discussed, as the use of the Internet is relevant and also in the light of the recent COVID-19 pandemic, which called for the reconfiguration of the healthcare system in hybrid online-offline forms [79]. The use of telemedicine, for example, is described as an ally able to guarantee continuity of care and quality of life to patients [80]. For this reason, the use of technology to engage patients in the educational interventions should be encouraged in order to overcome possible barriers. 

With regards to the quality of included studies, a consideration should be done when interpreting the findings. It should be acknowledged that in the QCC quality assessment checklist, the validity question concerning the full description of the adopted intervention and comparison (i.e., Were intervention/therapeutic regimens/exposure factor or procedure and any comparison(s) described in detail? Were intervening factors described?) has a significant weight on the assessment of the included studies since most of them were rated negatively in this domain. 

To conclude, given the mutated health needs of diabetic patients, the increasing burden of chronic disease on health systems, and the necessity of proper communication flows with respect to the past years, the present findings suggest that the research is struggling to bridge this gap in type 2 diabetes management. Food literacy and patient engagement should be considered as strongly related to patients’ care and should be assessed with validated measures in order to fine-tune the intervention and obtain more efficient results. In addition, the conceptualization of patient engagement should turn to considering a broader involvement of the patients not only in terms of self-management but also increasing their psychological engagement in all the care process. In doing so, disease management should be considered as a real lifestyle change, and in these terms, it demands that the patients not only to be instructed with information but also with appropriate tools that allow them to become an active partner of the care process. With this aim, web tools could be an enabler to facilitate this process by guaranteeing continuity of care and to actively involve patients but also to enhance professional exchange, which is relevant in chronic disease management. 

The present systematic review has strengths and limitations. It was conducted according to widely used methodological frameworks, such as PRISMA guidelines for the collection analysis and the QCC-validated quality checklist, which guaranteed the rigor of the results. However, due to the heterogeneity of the adopted measurement tools and variables, it was not possible to conduct a meta-analysis. Additionally, we included a broad range of studies, which may limit the review’s design. Nevertheless, wider inclusion of studies is needed since, sometimes, RCT is not the most suitable design for literacy and engagement interventions. 

In addition, differently from other recent reviews on the same population focusing particularly on one outcome (e.g., glycemic control) [81], the present systematic review took into account several outcomes. Although it was impossible to evaluate the efficacy of the individual studies’ features on the outcome assessment (e.g., glycated hemoglobin), in our review, we proposed a taxonomy of the main conceptualization of patient engagement with relative theoretical frameworks, which can be used to guide health policies for public health practitioners and decision makers. To do so, future studies are encouraged to use validated tools to measure both literacy and engagement in order to allow other researchers to compare the effectiveness of the results. Further studies investigating whether the several definitions of food literacy align with more nuanced understandings of food literacy, as reported in the scientific literature [82], are needed. Moreover, future researches providing a structured understanding of food literacy are imperatively required.

Besides, additional researches adopting technologies and, consequently, assessing their effects on outcomes are essential since, to date, it has been proven to result in relative utility and efficacy in patients’ education. 

## 5. Conclusions

In the current systematic review, we reviewed and synthesized the literature on interventions for food literacy and patient engagement targeting patients with type 2 diabetes. The review identified that, to date, interventions are heterogeneous in the adopted methodology, measures, and outcomes considered. The majority of the scientific debate focused both on literacy and engagement aspects, the latest mainly conceptualized as self-management. The majority of the studies involved a multidisciplinary team. Indeed, addressing food literacy in multidisciplinary diabetes educational and management programs improves important health outcomes. In addition, we found that the support materials used the most are paper-based or video; however, even if considered less to date, technology tools (e.g., social media, web sites, and apps) should be encouraged to stay in touch with the patients and to enhance their involvement in the educational intervention. The results presented in the review suggested some recommendations and practical implications based on the synthesis of current scientific debate. However, high-quality, randomized controlled trials and longitudinal studies are lacking and need to be conducted to verify the efficacy of these insights. 

## Figures and Tables

**Table 1:** Summary of the selected studies in the current systematic review.

ACY	Study Design	Exposure Timing	Outcomes Cathegory	N	Age Intervention (Mean, SD)	Age Control (Mean, SD)	Synthetic Results
Glasgow, R.E., USA, 2003	Rct	NR	clinical, behavioral, psychological, literacy	320	59; 9.2	NR	Improvements on behavioural, psychological, and biological outcomes. Difficulties in maintaining website usage over time.
Glasgow, R.E., USA, 2006	Rct	NR	clinical, behavioral, psychological, literacy	301	62.0 (11.7)	61.0 (11.0)	Reduction of dietary fat intake and weight.
							Among patients having elevated levels of HbA1c or lipids or depression at baseline, promising trend but not significant.
Petkova, V.B., Bulgaria, 2006	Pre-post study	NR	clinical, behavioral, psychological, literacy	24	64.96 (10.18)	NR	Improvement in patients’ diabetes knowledge and quality of life. Decreased frequency of hypo- and hyperglycemic incidents.
Song, M., Korea, 2009	Rct	2 days program	clinical	49	51.0 (11.3)	49.5 (10.6)	Reduction of mean HbA1c levels by 2.3% as compared with 0.4% in the control group. Increased adherence to diet.
Lujan, J., USA, 2007	Rct	8 weekly 2 h group sessions	clinical, psychological, literacy	150	58	NR	No significant changes at the 3-month assessment. At 6 months, adjusting for health insurance coverage, improvement of the diabetes knowledge scores and reduction of the HbA1c levels. The health-belief scores decreased in both groups.
Hill-Briggs, F., USA, 2008	Rct	90 min	literacy	30	60.9 (8.9)	62.1 (11.2)	Knowledge scores increased for below average (BA) and average (A) literacy groups. The BA group showed the largest gains in knowledge about recommended ranges for HbA1c, HDL cholesterol, and goals for CVD self-management. In the A group, the largest gains were found in differentiating LDL as “bad” cholesterol and knowing the recommended range for blood pressure.
Wallace, A.S., USA, 2009	Quasi-experimental	NR	behavioral, psychological, literacy	250	56	NR	Improvements (similar across literacy levels) in activation, self-efficacy, diabetes-related distress, self-reported behaviors, and knowledge.
Hamuleh, M., Iran, 2010	Rct	40 min	psychological and literacy	128	NA	NA	Using health-belief models for an educational intervention significantly modified benefits and barriers of perception to diet.
Hill-Briggs, F., USA, 2011	Rct	NR	clinical, behavioral, psychological, literacy	56 (29 intensive intervention; 26 condensed intervention)	61.1 (11.0)	61.5 (10.9)	Program scored as helpful and easy to understand. At immediate post intervention, participants in both programs demonstrated knowledge gain. At 3 months post intervention, only the intensive intervention was effective in improving knowledge, problem-solving skills, self-care, and HbA1c levels.
Carter, E.L., USA, 2011	Rct	30 min biweekly	clinical	47	52	49	Improvement in health outcomes and responsibility for self-health together with “other benefits’’.
Osborn, C.Y., USA, 2011	Rct	expected to be completed in 5 days	clinical, behavioral, psychological, literacy	118	56.7 (10.1)	NR	At 3-months: increased level of participants reading food labels and improvement in adherence to diet recommendations. No significant differences between the two groups on adjusted group means for physical activity and HbA1c levels.
Taghdisi, M.H., Iran, 2012	Quasi-experimental case-control study	20–30 min	psychological	78	49	NR	No significant increase in the mean score of quality of life. Significant differences in physical health, self-evaluation of quality of life, and self-assessment of health.
Castejón, A.M., USA, 2013	Rct	half a day session + 2 × 60 min consultation	clinical	43	55 (10)	54 (9)	Greater BMI and HbA1c levels reduction. No significant difference in blood glucose, blood pressure, or lipid levels.
Swavely, D., USA, 2014	Pre-post study	13 h	clinical, behavioral, psychological, literacy	106	56.8 (10.4)	NR	Significant improvements in diabetes knowledge, self-efficacy, and three self-care domains, such as diet, foot care, and exercise. At 3 months, levels of HbA1c decreased. No significant improvements in the frequency of blood glucose testing.
Calderón, J.L., USA, 2014	Rct	13 min video	literacy	240	NA	NA	No differences in the increase of DHLS scores occurred in both groups, but when adjusting for baseline DHLS score, sex, age, and insurance status, intervention group performed better. For participants with inadequate literacy levels, health literacy scores significantly increased.
Koonce, T.Y., USA, 2015	Rct	NR	literacy	128	54 (12.1)	53 (9.6)	DKT results at 2 weeks showed better performance on all literacy domains.
Kim, M.T., USA, 2015	Rct	weekly 2 h sessions × 6 weeks	clinical, behavioral, psychological, literacy	209	59.1 (8.4)	58.3 (8.5)	At 12 months: reduction in HbA1c levels and improvement in diabetes-related self-efficacy and quality of life.
Ichiki, Y., Japan, 2016	Pre-post study	20 min sessions	clinical	35	73.5 (12.2)	NR	Education was effective in participants with high baseline HbA1c levels (>8%) and poor understanding of their treatment.
Protheroe, J. UK, 2016	Rct	NR	clinical, behavioral, psychological, literacy	76	64.7 (11.2)	61.5 (10.1)	Participants in the LHT arm had significantly improved mental health and illness perception. The intervention was associated with lower resource use, better patient self-care management, and better QALY profile at 7-month follow-up.
Bartlam, B. UK, 2016	Rct	NR	literacy	40	43	NR	The intervention was acceptable to patients and, additionally, it resulted in behaviour changes.
Hung, J.Y., Taiwan, 2017	Quasi-experimental	1.5 h × 7 weeks	clinical, behavioral, psychological, literacy	95	61.3 (8.0)	58.5 (9.1)	Improvement in coping with disease and enhancement in self-care ability and positive effects on biochemical parameters, such as BMI, FPG, and HbA1c. DCMP could effectively increase the frequency of weekly SMBG and the DM health literacy levels among Taiwanese DM patients. No significant changes in depressive symptoms.
Lee, S.J., Korea, 2017	Rct	1 h	clinical, behavioral, psychological, literacy	51	74.5 (4.8)	74.5 (4.8)	Significant differences in DSK, DSE, DSMB, DHB, and HbA1c levels.
Wan, E.Y.F., Hong Kong, 2017	Quasi-experimental	NR	psychological	1039	63.80 (10.61)	68.54 (10.14)	RAMP-DM was more effective in improving the physical component of HRQOL, patient enablement, and general health condition in patients with suboptimal HbA1c than those with optimal HbA1c. However, the hypothesis that the RAMP-DM can improve HRQOL cannot be fully supported by these research findings.
Lee, M.-K., USA, 2017	Rct	NR	clinical	198	54.6 (9.7)	56.4 (8.7)	An increased SMBG frequency (twice a day) for the first 6 weeks with the telemonitoring device was associated with improved glycemic control (HbA1c and fructosamine blood levels) at 6 months.
Siaw, M.Y.L., Malaysia, 2017	Rct	20–30 min	clinical, behavioral, psychological, literacy	330	59.2 (8.2)	60.1 (8.1)	At 6 months: reduction of mean HbA1c, higher in patients with uncontrolled glycemia at baseline. Improvements in PAID and DTSQ scores, reduction in physician workload, and an average cost savings were observed.
		Every 4 to 6 Weeks					
Vandenbosch, J., Belgium, 2018	Pre-post study	NR	clinical, behavioral, psychological, literacy	366	62.1 (11.99)	62.5 (11.12)	Positive effects of DSME programmes on self-reported self-management behaviours and almost all psychological and health outcomes regardless of HL level. Individual and group-based programs performed better than self-help groups.
Kim, S.H., Korea, 2019	Rct	NR	clinical, behavioral, psychological	155	NR	NR	At 9 weeks, patients with high HL showed higher levels of patient activation than those with low HL in the control group, while the difference related to HL was no longer significant in intervention groups. At 9 weeks, patients who received the telephone-based, HL-sensitive diabetes management intervention had a significantly higher score for self-care behaviors. No significance on HbA1c levels.
Rasoul, A.M., Iran, 2019	Rct	90′ session 3 times a week	psychological	98	31.36 (5.29)	32.98 (4.42)	Significant differences both in anthropometric variables/metabolic indicators (waist circumference, FBS, BMI) and quality of life score.
Cheng, L. China, 2019	Rct	NR	psychological	242	56.13 (10.72)	53.9 (13.01)	At one week, significant improvements on empowerment level, reduction in terms of emotional-distress, regimen-distress, and physician-related distress was observed. Empowerment, emotional-distress, and improvement in quality of life were found to be still significant at 3 months.
McGowan, P., Canada, 2019	Pre-post study	30 min	clinical, behavioral, psychological, literacy	115	60.8 (9.3)	NR	At 12 months: reduction of HbA1c level, fatigue, and depression level; improvement of general health, activation, empowerment, self-efficacy, and increased communication with physician.
Hernández-Jiménez, S., Mexico, 2019	Pre-post study	sessions 30–60 min	clinical	1837	51.1 (10.3)	NR	At 4 months, positive effects on empowerment, HL, anxiety, depression, quality of life, HbA1c levels, BP, and LDL. Decreasing trends were also observed at 12 months.
Sims Gould, J., Canada, 2019	Pre-post study	NR	behavioral, literacy	17	NR	NR	The GMVs increased participants’ diabetes literacy and self-management skills
White, R.O., 2021	Rct	NR	behavioral, literacy, clinical, psychological	364	51 (36–60)	50 (37–60)	At 12 months: decreased risk of poor eating and better treatment satisfaction, self-efficacy, and HbA1c levels.

BMI, body mass index; CVD, cardiovascular disease; DCMP, diabetes conversation map program; DHLS, diabetes health literacy scale; DKT, diabetes knowledge test; DM, diabetes mellitus; DSE, diabetes support and education; DSK, diabetes self-management knowledge; DSME, diabetes self-management education; DTSQ, diabetes treatment satisfaction questionnaire; FBS, fasting blood sugar; FPG, fasting plasma glucose; HbA1c, glycated hemoglobin; HDL, high density lipoprotein; HL, health literacy; HRQOL, health-related quality of life; LDL, low density lipoprotein; LHT, lay health trainer; NA, not available; NR, not reported; PAID, problem areas in diabetes; QALY, quality-adjusted life year; RAMP-DM, risk assessment and management program-diabetes mellitus; Rct, randomized controlled trial; SMBG, self-monitoring of blood glucose.

**Table 2:** Quality assessment attributes for each quantitative study included in the current systematic review, assessed by the Academy of Nutrition and Dietetics’ Quality Criteria Checklist.

Author (year)	1	2	3	4	5	6	7	8	9	10	Overall
Glasgow, R.E. (2003)	Y	Y	Y	N	N	N	Y	Y	Y	Y	**0**
Kim, M.T. (2015)	Y	Y	Y	N	N	N	Y	Y	Y	Y	**0**
Rasoul, A.M. (2019)	Y	Y	Y	Y	N	N	Y	Y	N	Y	**0**
Cheng, L. (2019)	Y	Y	Y	U	Y	Y	Y	Y	Y	Y	**+**
Protheroe, J. (2016)	Y	Y	Y	Y	Y	N	Y	Y	Y	Y	**0**
Bartlam, B. (2016)	Y	Y	Y	U	Y	N	Y	Y	Y	Y	**0**
Lujan, J. (2007)	Y	Y	N	U	N	N	Y	Y	N	Y	**0**
Lee, S.J. (2017)	Y	Y	Y	Y	Y	Y	Y	Y	Y	Y	**+**
Hill-Briggs, F. (2011)	Y	Y	N	Y	N	N	Y	Y	Y	Y	**0**
Kim, S.H. (2019)	Y	Y	Y	Y	N	N	Y	Y	Y	Y	**0**
Glasgow, R.E. (2006)	Y	N	Y	Y	N	N	Y	Y	Y	Y	**0**
Hamuleh, M. (2010)	Y	N	Y	N	N	Y	Y	Y	Y	Y	**0**
Lee, M.K. (2017)	Y	U	Y	U	N	N	Y	Y	Y	Y	**0**
Siaw, M.Y.L. (2017)	Y	N	Y	N	N	Y	Y	Y	Y	Y	**0**
Calderón, J.L. (2014)	Y	N	Y	U	Y	N	Y	Y	Y	Y	**0**
Song, M. (2018)	Y	N	Y	U	N	N	Y	Y	Y	Y	**0**
Castejón, A.M. (2013)	Y	Y	Y	N	N	Y	Y	Y	Y	Y	**+**
Carter, E.L. (2011)	Y	Y	Y	N	N	Y	Y	Y	Y	Y	**+**
Koonce, T.Y. (2015)	Y	Y	Y	N	N	Y	Y	Y	Y	Y	**+**
Osborn, C.Y. (2011)	Y	Y	Y	N	N	N	Y	Y	N	Y	**0**
Hill-Briggs, F. (2008)	Y	N	Y	N	N	Y	Y	Y	Y	Y	**0**
Wan, E.Y.F. (2017)	Y	N	N	N	N	N	Y	Y	Y	Y	**0**
Hung, J.Y. (2017)	Y	Y	Y	U	N	Y	Y	Y	Y	Y	+
Wallace, A.S. (2009)	Y	Y	NA	N	N	N	Y	Y	N	Y	**0**
Taghdisi, M.H. (2012)	Y	Y	NA	N	N	N	Y	Y	Y	Y	**0**
Vandenbosch, J. (2018)	Y	Y	NA	N	N	N	Y	Y	Y	Y	**0**
Hernández, J.S. (2019)	Y	Y	NA	N	N	N	Y	Y	Y	Y	**0**
Swavely, D. (2014)	Y	U	NA	U	N	Y	Y	Y	Y	Y	**0**
Petkova, V.B. (2006)	Y	Y	NA	N	N	N	Y	Y	N	Y	**0**
Sims, G.J. (2019)	Y	Y	NA	N	N	N	Y	Y	Y	Y	**0**
McGowan, P. (2019)	Y	Y	NA	N	N	N	Y	Y	Y	Y	**0**
Ichiki, Y. (2016)	Y	Y	NA	U	N	Y	Y	Y	Y	Y	**+**
White, R.O., 2021	Y	Y	Y	Y	Y	U	Y	Y	Y	Y	**+**

Y, yes; N, no; NA, not applicable; U, unclear; +: the report has clearly addressed issues of inclusion/exclusion, bias, generalizability, and data collection and analysis.

**Table 3:** Summary of interventions’ outcomes and measures used in the selected studies.

ACY	Outcome Categories and Measure Tools
Glasgow, R.E., USA, 2003	Clinical: Glycated hemoglobin (HbA1c) determination was based on turbidimetric immunoinibition using hemolized whole blood, with the Hitachi 717; Block/NCI Fat Screener scale Behavioral: Kristal Fat and Fiber Behavior Psychological: Diabetes Support Scale; Center for Epidemiologic Studies–Depression scale Literacy: American Diabetes Association Provider Recognition Program
Glasgow, R.E., USA, 2006	Clinical: Block/NCI Fat Screener scale; National Glycohemoglobin Standardization Program (NGSP) Roche methodologies; enzymatic methods Behavioral: NCI Fruit and Vegetable Screener Psychological: Diabetes Distress Scale; Patient Health Questionnaire Literacy *
Petkova, V.B., Bulgaria, 2006	Clinical * Behavioral * Psychological: Diabetes Questionnaire Literacy *
Song, M., Korea, 2009	Clinical: HbA1c levels were measured using a high-performance liquid chromatography technique with a Variant II analyzer (Bio-Rad, Montreal, QC, Canada) Behavioral: The self-report questionnaire on adherence
Lujan, J., USA, 2007	Clinical: Glycemic control for HbA1c levels was measured by a finger-stick procedure to obtain the blood and a Bayer 2000 analyzer to analyze the sample Psychological: Bilingual Diabetes Health-Belief Model Literacy: Bilingual Diabetes Knowledge Questionnaire
Hill-Briggs, F., USA, 2008	Literacy: Wide-Range Achievement Test
Wallace, A.S., USA, 2009	Behavioral: Patient Activation Measure Psychological: Diabetes Distress Scale Literacy: Test of Functional Health Literacy for Adults
Hamuleh, M., Iran, 2010	Clinical: HbA1c was measured by using photometer method with Biochemical autoanalyzer device model BT3000 Psychological * Literacy *
Hill-Briggs, F., USA, 2011	Clinical: HbA1c was measured using high-pressure liquid chromatography; LDL and HDL were measured using standard techniques; blood pressure was assessed using a random-zero sphygmomanometer Behavioral: Summary of Diabetes Self-Care Activities scale Psychological: Test Health Problem-Solving Scale Literacy: Diabetes and Cardiovascular Disease Knowledge
Carter, E.L., USA, 2011	Clinical *
Osborn, C.Y., USA, 2011	Clinical * Behavioral * Psychological * Literacy *
Taghdisi, M.H., Iran, 2012	Psychological: The World Health Organization quality-of-life assessment
Castejón, A.M., USA, 2013	Clinical *
Swavely, D., USA, 2014	Clinical * Behavioral: Summary of Diabetes Self-Care Activities tool Psychological: Stanford Diabetes Self-Efficacy Literacy: Short Test of Functional Health Literacy in Adults; Spoken Knowledge in Low Literacy Patients with Diabetes
Calderón, J.L., USA, 2014	Literacy: Functional Health Literacy in Adults; Diabetes Health Literacy Survey (DHLS)
Koonce, T.Y., USA, 2015	Literacy: Modified version of the Michigan Research and Training Center’s Diabetes Knowledge Test; modified version of the Subjective Numeracy Scale
Kim, M.T., USA, 2015	Clinical * Behavioral: Summary of Diabetes Self-Care Activities scale Psychological: Stanford Chronic Disease Self-Efficacy scale; Diabetes Quality-of-Life Measure (DQOL) Literacy: Diabetes Knowledge Test (DKT)
Ichiki, Y., Japan, 2016	Clinical: HbA1c levels were determined according to the National Glycohemogloblin Standardization Program (NGSP)
Protheroe J., UK, 2016	Behavioral: Summary of Diabetes Self-Care Activities Measure Psychological: Diabetes Quality of Life Brief Clinical Inventory for Quality of Life; EQ5D for health-related Quality of Life; Warwick-Edinburgh Mental Well-Being; Brief Illness Perception Score; Quality of Life SF12 Literacy: Newest Vital Sign U
Bartlam B.,UK, 2016	Psychological: Warwick-Edinburgh Mental Well-Being Scale Literacy: Newest Vital Sign UK
Hung, J.Y., Taiwan, 2017	Clinical * Behavioral * Psychological: Taiwanese Depression Questionnaire Literacy *
Lee, S.J., Korea, 2017	Clinical: HbA1c, blood pressure, and serum lipids Behavioral: The Korean version of the Summary of Diabetes Self-Care Activities Questionnaire Psyhological: Health Belief Scale for Diabetes; The Diabetes Management Self-Efficacy Scale for Older Adults Literacy: Korean Health Literacy Assessment Tool; Diabetes Self-Management Knowledge; The Diabetes Self-Management Knowledge for Older Adults
Wan, E.Y.F., Hong Kong, 2017	Psychological: Quality of Life SF-12v2; Patient-Enablement Instrument
Lee, M.-K., USA, 2017	Clinical: HbA1c level; fructosamine, weight, blood pressure, and LDL cholesterol were measured by Samsung Health Diary (SHD) telemonitoring device
Siaw, M.Y.L., Malaysia, 2017	Clinical * Behavioral * Psychological: Problem Areas in Diabetes Questionnaire; Diabetes Treatment Satisfaction Questionnaire Literacy *
* Vandenbosch, J., Belgium, 2018	Behavioral: Summary of Diabetes Self-Care Activities Questionnaire Psychological: Problem Areas in Diabetes Questionnaire; SF-36 Health survey Literacy: Appraisal of Diabetes Scale (ADS)
Kim, S.H., Korea, 2019	Clinical * Behavioral: Patient Activation Measure; Revised Korean version of the Summary of Diabetes Self-Care Activities measure Psychological * Literacy: Short Form of the Korean Functional Health Literacy Test
Rasoul, A.M., Iran, 2019	Psychological: Diabetes Quality of Life Measure
Cheng, L. China, 2019	Psychological: Diabetes Empowerment Scale-Short Form; Diabetes Distress Scale; Audit Diabetes Dependent Quality of Life
McGowan, P., Canada, 2019	Clinical * Behavioral: Patient Activation Measure; Morisky Medication Adherence Scale Psychological: Self-Efficacy scale; Patient Health Questionnaire-9; Diabetes Empowerment Scale Literacy *
Hernández-Jiménez, S., Mexico, 2019	Clinical: Fasting concentrations of glucose, creatinine, lipids and HbA1c (Bio-Rad Variant II Turbo HbA1c Kit 2, with HPLC method) were assessed in each visit; Albuminuria/creatinuria ratio (ACR) (SYNCHRON CX system with colorimetric method); body composition was assessed by bioimpedance (body composition analyzer JAWON medical ioi353). Behavioral: National Committee for Quality Assurance criteria for the achievement of treatment goals; International Physical Activity Questionnaire Psychological: The Diabetes Empowerment Scale-Short Form; Hospital Anxiety and Depression Scale; Diabetes Quality-of-Life Measure; Problem Areas in Diabetes Questionnaire Literacy: Diabetes Knowledge Scale
Sims Gould, J., Canada, 2019	Behavioral * Literacy *
White, R.O., 2021	Clinical * Behavioral: Summary of Diabetes Self-Care Activities; Adherence to Refills and Medication Scale; Personal Diabetes Questionnaire Psychological: Diabetes Treatment Satisfaction Questionnaire; Perceived Diabetes Self-Management Scale Literacy: Short Test of Functional Health Literacy in Adults; Diabetes Numeracy Test (DNT)

* No reported measurement tool. NCI, National Cancer Institute; LDL, low-density lipoprotein; HDL, high-density lipoprotein.

## Abbreviations

ADA	American Diabetes Association
PICO	Population, Intervention, Comparator, Outcome
PRISMA	Preferred Reporting Items for Systematic Reviews and Meta-analyses
AHRQ	Agency for Healthcare Research and Quality
RCT	Randomized Controlled Trial
PRECEDE	Predisposing, Reinforcing, and Enabling Constructs in Education/environmental Diagnosis and Evaluation
IMB	Information–Motivation–Behavioral
DOIMP	Diabetes Outpatient Intensive Management Program
DSMOF	Diabetes Self-Management Outcome Framework
